# Angiotensin System Inhibitors May Improve Outcomes of Patients With Castration-Resistant Prostate Cancer During Abiraterone Acetate Treatment—A Cardio-Oncology Study

**DOI:** 10.3389/fonc.2021.664741

**Published:** 2021-04-01

**Authors:** Michał Wilk, Anna Waśko-Grabowska, Iwona Skoneczna, Sebastian Szmit

**Affiliations:** ^1^ Department of Oncology, Centre of Postgraduate Medical Education, European Health Centre, Otwock, Poland; ^2^ Maria Sklodowska‐Curie National Research Institute of Oncology, Warsaw, Poland; ^3^ Department of Pulmonary Circulation, Thromboembolic Diseases and Cardiology, Centre of Postgraduate Medical Education, European Health Centre, Otwock, Poland

**Keywords:** prostate cancer, abiraterone, cardiovascular, angiotensin system inhibitor, cardio-oncology

## Abstract

**Background:**

Abiraterone acetate (ABI) therapy improves overall survival in metastatic prostate cancer (PC) patients; however, this effect may be diminished by concurrent comorbidities. We aimed to evaluate the influence of pre-existing chronic diseases and concomitant medications on the course of ABI treatment among post-chemotherapy patients with metastatic castration-resistant prostate cancer patients (mCRPC).

**Methods:**

From the Polish National Health Fund database, we identified 93 post-chemotherapy, mCRPC patients, who were qualified for ABI treatment in our oncology center between 2014 and 2018. Survival curves and Cox proportional hazard models (univariate and multivariate) were used to determine the predictors for longer time to treatment failure (TTF) of ABI therapy.

**Results:**

Median TTF was 9,8 months (IQR: 0,6–56,5) Factors associated with longer TTF were: well controlled hypertension (HR, 0.59; 95% CI. 0.38–0.90; p = 0.02), stable coronary artery disease (HR, 0.56; 95% CI, 0.33–0.95; p=0.03), the use of angiotensin system inhibitor (ASi) (HR, 0.61; 95% CI 0.4–0.94; p = 0,02). Patients who were receiving ASi had median TTF of 12.2 months versus 5.8 months in men who did not receive ASi before ABI initiation. At the start of ABI therapy, the aforementioned groups did not differ in terms of well-known prognostic factors: Gleason score, PSA level, or the number of patients with visceral metastases. In a multivariate analysis, the use of ASi remained statistically significant, even after adjustment for well-known oncological factors (HR, 0.57; 95% CI, 0.34–0.98; p = 0.04).

**Conclusions:**

The use of ASi may enhance and prolong ABI therapy in post-docetaxel mCRPC patients and may potentially be considered a new, non-oncological, predictive factor for longer TTF. This association requires a prospective validation.

## Introduction

Prostate cancer (PC) is the most common cancer site diagnosed in men in Europe and USA (1, 2). Over the last decade the therapy of metastatic disease has been rapidly evolving and numerous new agents which improve overall survival have been developed. One of such important change was the approval of abiraterone acetate (ABI). It is the second-generation antiandrogen which blocks the synthesis of androgens *via* inhibition of CYP17A enzyme and therefore impedes prostate cancer progression (3). Despite relatively low prevalence of severe adverse events in registration trials, a growing concern is arising regarding the safety of this drug especially among those with cardiovascular (CV) comorbidities, since the drug can induce or exacerbate hypertension, coronary artery disease, rhythm disturbances or heart failure (4). There is still an ongoing debate which group of patients may benefit most from ABI therapy, therefore, the research of potential clinical factors of predictive and prognostic value is essential.

The impact of CV diseases on the course of PC is an important issue, since they are the most common comorbidities and the second leading cause of mortality in this population (5, 6). In addition, PC shares many similar risk factors that are responsible for the development of cardiac or vascular diseases, which may explain their high co-occurrence rate (7). Many individuals with PC receive numerous agents for the treatment of co-existing CV comorbidities (e.g., lipid-lowering drugs, antiplatelet therapy, antihypertensives or glucose-lowering agents). This concurrent use with a systemic anti-cancer therapy may presumably have an influence on its tolerance and even effectiveness.

In our study we evaluated the influence of comorbidities and concomitant medications on the course of ABI treatment among men with metastatic castration resistant prostate cancer (mCRPC).

## Materials and Methods

### Overview

The study provides the analysis of data from the Polish National Health Fund Drug Program database and the European Health Centre Otwock hospital’s records of mCRPC patients receiving ABI with prednisone who progressed after chemotherapy with docetaxel, and who were qualified for this treatment in our oncology center in the period between 2014 and 2018. The treatment consisted of abiraterone acetate (Zytiga^®^, Janssen) 1000 mg once a day with 10 mg of oral prednisone. Every patient signed informed consent for the treatment and data collection. Thee protocol of the study was approved by Bioethical Committees at the Centre of Postgraduate Medical Education in Poland (Resolution Number 83/PB/2016) and was in accordance with the 1964 Helsinki declaration and its later amendments or comparable ethical standards.

### Patients

In Poland, the use of ABI is regulated by the Drug Program dedicated to mCRPC patients. Inclusion and exclusion criteria were specified by the National Health Fund Department.

Every Patient had a pathological diagnosis of prostate adenocarcinoma, had a radiologic evidence of metastases, and had received docetaxel therapy before ABI initiation. The disease progression after docetaxel was defined as if a patient had two consecutive increases in the prostate specific antigen (PSA) concentration (from the lowest PSA level reached during or after docetaxel) or radiographic evidence of disease progression in bone or soft tissue with or without the rise of PSA value post chemotherapy. All Men received androgen deprivation therapy (surgical or pharmacological), with a serum testosterone level of 50 ng/dl or less (≤1.7 nmol/L) and had an Eastern Cooperative Oncology Group (ECOG) performance status score of 0 or 1 (with “0” indicating that the patient is fully active and able to carry on all pre-disease activities without restriction and “1” indicating that the patient is restricted in physically strenuous activity but is ambulatory and able to carry out work of a light or sedentary nature (8). Patients had no significant hepatic dysfunction (only Child-Pugh class A was eligible). Unstable or uncontrolled cardiac disorders were not allowed as well. None of patients had a history of prior abiraterone acetate, enzalutamide or ketoconazole therapy. Patients’ characteristics were registered before the initiation of ABI treatment.

### Clinical Measurements and End Point

The primary endpoint of our analysis was the time to treatment failure (TTF), described as the time period between the initiation of ABI to the moment of its termination (defined as the cancer disease progression, unaccepted toxicity, hypersensitivity to the drug or the patient’s death).

Disease progression was defined as

- the occurrence of at least two of the following three types of progression in total:
  1) clinical, defined as pain progression (inclusion of a new opioid for more than 2 weeks), or the occurrence of skeletal related events, or deterioration of the patient’s performance status to at least Grade 2 (according to the WHO classification),
  2) biochemical, defined as PSA progression (three consecutive increases in PSA, measured at least in weekly intervals, with proven increases of at least 50% from ABI baseline),
  3) radiological, defined as the appearance of at least two new metastatic lesions, confirmed by scintigraphy.
- Response Evaluation Criteria In Solid Tumors ver. 1.1 were met (regardless of other types of progression mentioned above).

As potential factors influencing TTF several characteristics were analyzed:

- Gleason score, metastases location, initial PSA and testosterone level at ABI therapy initiation;
- Age, body-mass index (BMI),
- Medical history including co-existing CV diseases (i.e., arterial hypertension, coronary artery disease, dyslipidemia, atrial fibrillation, cerebral stroke) and diabetes;
- Type and number of concomitant medications, e.g., b-blockers, lipid-lowering drugs (statins), hypoglycemic medications (metformin) and with particular emphasis on angiotensin system inhibitor (ASi), i.e., angiotensin-converting enzyme inhibitors (ACE-I), angiotensin receptor blockers (ARB).

### Statistical Analysis

Statistical analyzes were performed using Stata^®^ Software ver. 14.1 (StataCorp LLC). Nominal parameters were presented as a percentage frequency. The χ^2^ test for categorical variables, the independent t-test for continuous, normally distributed variables and the Mann-Whitney U for non-normally distributed variables were used for comparisons between the groups. The r-Pearson and Spearman correlation were used for assessing the association between continuous variables. Survival curves and Cox proportional hazard model (univariate and multivariate) were used to determine the predictors for longer TTF during ABI treatment. A level p<0.05 was recognized as statistically significant.

## Results

According to the Polish National Health Fund Drug Program database, 93 patients were qualified for ABI therapy (mCRPC patients who received prior docetaxel therapy) in our hospital between January 2014 and December 2018. Till December 31, 2020, four men were still receiving ABI (4.3%). The baseline characteristics of all the patients are summarized in **Table 1**.

**Table 1:** Characteristics of the patients at baseline.

Characteristics	Value
**Age**	
Median (IQR)—years	69 (43–88)
>=70 yr—n (%)	45 (48%)
**BMI**	
Mean ± SD	27.7 ± 3,81
**PSA [ng/mL]**	
Median (IQR)	87.7 (0,3–5 130)
**Gleason score**	
Median (IQR)	8 (5–10)
>=8—n (%)	54 (58%)
Missing data—no. (%)	7 (8%)
**Testosterone [ng/dl]**	
Median (IQR)	28.5 (1–50)
**Medications**	
Median number of drugs taken—n. (IQR)	2 (0–9)
>3 drugs—n (%)	33 (35%)
**Location of metastases**	
Bone only—n (%)	40 (43%)
Visceral metastases—n (%)	53 (57%)
**Purpose of ABI termination**	
Disease progression—n (%)	59/89 (66%)
Death—n (%)	20/89 (22%)
Other*—n (%)	11/89 (12%)

*Defined as unacceptable toxicity, patient’s resignation or lost to follow-up for unknown reason.

Overall, median TTF was 9.8 months (IQR, 0.6–56.5). TTF significantly correlated with Gleason score (r = −0.344; p<0.01), and initial PSA level (r = −0,53; p < 0.01). We did not find any significant correlation between TTF and testosterone level (p = 0.19), BMI (p = 0.07), or the number of concomitant medications taken (p = 0.09).

The analysis of the influence of comorbidities on TTF is presented in **Table 2**.

**Table 2:** The prevalence of comorbidities and their impact on TTF.

Comorbidity	Patients, n (%)Total = 93	Median TTF(without vs with comorbidity)[months]	HR (95%CI)	p value
Hypertension	39 (42%)	5.8 vs 12.0	0.59 (0.38–0.90)	0.02*
Coronary artery disease	20 (22%)	6.5 vs 12.3	0.56 (0.33–0.95)	0.03*
Atrial fibrillation	16 (17%)	9.8 vs 8.0	0.78 (0.44–1.39)	0.39
Diabetes	19 (20%)	6.5 vs 12.3	0.60 (0.36–1.02)	0.06
Dyslipidemia	12 (13%)	9.8 vs 10.4	0.67 (0.35–1.29)	0.23
Cerebral stroke	8 (9%)	9.8 vs 6.5	1.50 (0.72–3.12)	0.28
Obesity^#^	26 (28%)	8.2 vs 11.5	0.97 (0,61–1.54)	0.88

*Statistically significant; ^#^Defined as body mass index ≥ 25.

TTF, time to treatment failure; HR, hazard ratio; CI, confidence interval.

Patients who suffered from well-controlled hypertension or stable coronary artery disease had significantly longer TTF than those without such comorbidities (HR, 0.59; 95% CI, 0.38–0.90; p = 0.02 and HR, 0.56; 95% CI, 0,33–0,95; p = 0.03, respectively). There was also a positive but not significant trend for longer TTF in diabetic group (HR, 0.60; 95% CI, 0.36–1.02, p = 0.06).

The association between concomitant medications and TTF are presented in **Table 3**.

**Table 3:** Type of concomitant medications and their influence on TTF (univariate analysis).

Type of medication	Patients no. (%)Total = 93	Median TTF (with vs without medication) [months]	p value
Angiotensin system inhibitor	37 (40%)	12.2 vs 5.8	0.03*
B-blocker	36 (39%)	12.1 vs 7.0	0.29
Statin	23 (25%)	12.1 vs 8.2	0.22
Metformin	12 (13%)	9.9 vs 9.8	0.24

RAAS, renin-angiotensin-aldosterone system; TTF, time to treatment failure.

Out of 37 men who were receiving ASi, 35 (94.6%) individuals had hypertension and/or CAD. Other two men receiving ASi had a single diagnosis of heart failure.

**Figure 1:**
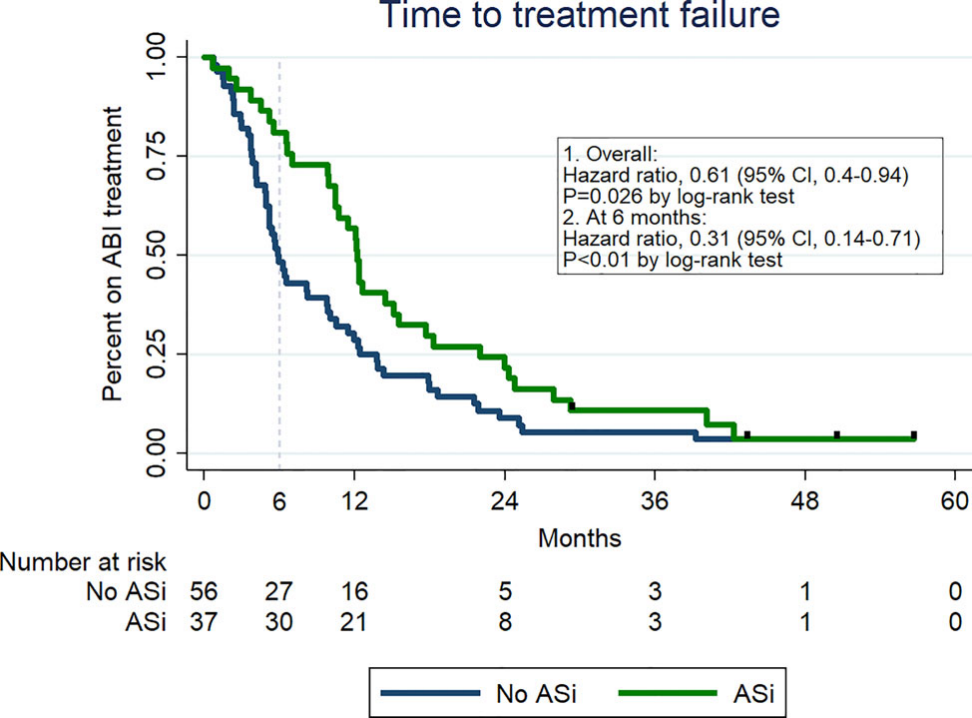
Kaplan-Meier curves of time to treatment failure depending on the ASi therapy.

From all types of medications, only the use of ASi correlated with TTF, which was significantly longer in “ASi group” versus those without such medications (12.2 months vs 5.8 months; HR 0,61; 95% CI, 0.4–0.94; p = 0.02). Kaplan-Meier curves are presented in **Figure 1**.

After 6 months of ABI therapy, 36 patients met at least one treatment failure criterium. In this group, there were 12 (33%) deaths, and 18 (50%) disease progressions. Six (17%) men stopped ABI due to other reasons (treatment toxicity, patient’s resignation or lost to follow-up). Among 36 patients who were not treated with ABI for more than 6 months, 27 individuals did not receive ASi versus 9 men who received this medications (HR for ABI discontinuation before 6 months: 0.31 (95% CI, 0.14–0.71; p < 0.01).

The comparison of two study populations (with or without ASi) is presented in **Table 4**.

**Table 4:** The comparison of the patients with or without ASi therapy.

Characteristics	No ASi therapy(n = 56)	ASi therapy (n = 37)	p value
Age, median (IQR)	67 (43–82)	72 (52–88)	<0.01*
>=70 years old—n (%)	21 (38%)	24 (65%)	<0.01*
Gleason score, median (IQR)	8 (6–10)	7 (5–9)	0.16
Initial PSA, median (IQR)	113.5 (0.3–5130)	76.7 (6.7–1000)	0.35
BMI, mean ± SD	27,1 ± 3,85	28.1 ± 3.63	0,07
Number of concomitant medications,^#^ median (IQR)	1 (0–8)	5 (1–9)	<0.01*
Metformin	3	9	<0.01*
B-blocker	15	21	<0.01*
Statin	7	16	<0.01*
Purpose of ABI termination:			
Death—n (%)	14 (25%)	6 (16%)	0.31
Disease progression—n (%)	32 (57%)	27 (72%)	0.12
Location of metastases:			
Bone metastases only—n (%)	23 (41%)	17 (46%)	0.67
Visceral metastases—n (%)	33 (59%)	20 (54%)	0.64

*Statistically significant.

ASi, angiotensin system inhibitor; IQR, interquartile range; PSA, prostate-specific antigen; BMI, body mass index.

Patients receiving ASi were statistically older (with more men aged >=70 years) and were generally receiving more concomitant medications that those without ASi. Two groups did not differ in terms of prognostic factors i.e., initial PSA level, and Gleason score (p>0.05).

After adjusting for oncological factors, the use of ASi remained predictive for longer TTF (HR, 0.57; 95% CI, 0.34–0.98; p = 0.04) (**Table 5**).

**Table 5:** Predictive factors determining time to treatment failure (multivariate analysis).

Characteristics	HR (95% CI)	p-value
Age (years)	1.01 (0.99–1.04)	0.250
Gleason score	1.25 (1.00–1.56)	0.046*
Initial PSA	1.00 (1.00–1.01)	<0.001*
Bone metastases	3.19 (0.94–10.8)	0.062
Visceral metastases	0.73 (0.46–1.17)	0.192
Testosterone	0.99 (0.97–1.00)	0.156
The use of ASi	0.58 (0.34–0.98)	0.042*

*Statistically significant.

PSA, prostate specific antigen; ASi, angiotensin system inhibitor; HR, hazard ratio; CI, confidence interval.

Median overall survival (OS) was 14.3 months (IQR, 0.7–63.5). A positive, but not statistically significant trend in overall survival was seen among patients who received ASi treatment (19.6 vs 10.8 months; HR for death, 0.68; 95% CI: 0.44–1.07; p = 0.09).

## Discussion

To our knowledge this is the first study presenting data of the positive effect of ASi on the course of ABI treatment in post-chemotherapy mCRPC patients. In our analysis we found that men receiving ASi had statistically longer TTF than those without such medications. It must be highlighted that there were no significant differences between these groups in terms of well-known prognostic factors, such as Gleason score, or initial PSA level before starting ABI treatment. Furthermore, ASi population was statistically older, with more patients older than 70 years, and received more concomitant medications. Despite this, those individuals still achieved longer TTF than those without ASi.

There are several possible explanations of such effect. Renin-angiotensin-aldosterone system (RAAS) consists of three major compounds: renin, angiotensin II, and aldosterone and their main role is to impact blood pressure, fluid volume, and sodium-potassium balance (9). In addition to its main regulatory function of arterial pressure and water/electrolyte homeostasis, it may also play a role in a cancer growth and progression (10), as the components of RAAS are present both in normal and neoplastic prostate tissue (11). In vitro studies on prostate cancer cell lines, inhibition of RAAS resulted in a decrease of angiogenesis, enhancement of apoptosis and thus demonstrated anti-metastatic activity (12, 13). Clinically, in a small sample study, the use of captopril resulted in a lower PSA recurrence after radical prostatectomy (14). The clinical interaction between RAAS and cancer disease, was also the subject of phase II pilot study, in which, heavily pretreated mCRPC patients received ARB (candesartan). Six men (26.1%) showed a PSA response, and a half of the patients presented an improved or stable performance status during follow-up (15). Furthermore, in recent study, the use of ARBs among PC patients, was associated with lower prostate cancer specific and overall mortality (16). Nevertheless, this study included heterogeneous group of patients e.g., with localized or metastatic disease, which could have an important impact on survival results. Taking all the above results into account, these data suggest that ASi may have an anti-neoplastic potential and they deserve more attention in terms of possible benefits to PC treatment.

Second, ABI impairs androgen biosynthesis by inhibition of steroid 17-hydroxylase/17,20-lyase (cytochrome P450c17) which induces several adverse hormonal changes, including a decrease in cortisol levels and a compensatory increase in adrenocorticotropic hormone (ACTH) (17). This adaptive rise of ACTH leads to accumulation of steroids with mineralocorticoid properties [e.g., aldosterone (18)] and eventually results in adverse clinical complications such as fluid retention, hypertension or hypokalemia (19). Cortisol deficiency and its endocrine consequences, which can be observed in patients receiving ABI, are inhibited by the mandatory oral administration of glucocorticosteroids. Our hypothesis is that the use of ASi decrease aldosterone serum level, and thus, reduce the mineralocorticoid-related toxicity of ABI. This in turn, may lead to a more tolerable ABI therapy.

Another possible mechanism may be related to the androgen axis. Androgen receptor activation by testosterone or its more potent metabolite, dihydrotestosterone, is crucial for PC growth and progression. Moreover, PC can synthesize androgens itself, which may explain the acquisition of castration resistance during androgen deprivation therapy (20). Targeting this mechanisms may result in a reduced cancer cell proliferation and disease stabilization (21). Among men with mCRPC, maintaining the testosterone at the lowest possible levels is associated with improved survival and better prognosis (22). RAAS may be involved in the regulation of a metabolism of male sex hormones, however data regarding this issue is limited. In several studies, testosterone serum levels were found to be significantly lower among men receiving ASi (23, 24). In contrast, however, there is also a study which does not support these findings (25). In our analysis, we did not find any correlation between the use of ASi and serum testosterone level. Importantly, there were no statistically significant differences in testosterone levels between ASi and no ASi group.

The course of anticancer treatment can be also affected by pre-existing comorbidities, especially those related to the CV system. Surprisingly, in several cancer types, CV diseases may have a positive effect in terms of clinical outcomes, e.g., a longer progression-free survival in patients with hypertension who are receiving cardiotoxic therapy for kidney cancer (26). Nevertheless, a recent large, retrospective analysis by Lu-Yao et al., reported that patients with PC and CV comorbidities experienced a higher short-term mortality (during the first 6 months) d ABI, than those without pre-existing CVDs (RR = 1.16; 95% CI, 1.00–1.36 for those with a single CV comorbidity, and RR = 1.56; 95% CI, 1.29–1.88 for men with three or more CV diseases) (27). Our findings are drawing a different landscape. Thirty-six patients had their ABI treatment terminated at 6 months period. Among those who did not receive ASi, the risk of ABI discontinuation was significantly higher than in the ASi group. This observation can be partially explained by the fact, drugs which block RAAS, play a protective role for the CV system (28). For example, both ACE-I or ARB reduce the risk of myocardial infarct, stroke, or CV-related death (29, 30). In animal and *in vitro* studies, the inhibition of the RAAS impairs plaque development and suppress atherosclerosis (31). Additionally, ASi may also reduce the risk of rhythm disturbances e.g., atrial fibrillation (32). Moreover, the prophylactic use of ASi as a prevention of CV complications showed promise in several cancer types e.g., before anthracycline treatment for breast cancer (33), among lymphoma (34), kidney (35), or pancreatic cancer patients (36).

In our study, we found that the use of ASi is associated with longer TTF, even after adjustment for well-known predictive, oncological factors (multivariate analysis included: age, initial PSA level, Gleason score, testosterone level, and the presence of bone and visceral metastases). The use of ASi may potentially be considered as a new, non-oncological, predictive indicator for longer ABI therapy.

The design of the current study is subject to limitations. First of all, this is a retrospective, single center study. Second, the sample size is small, therefore, limiting the number of factors included in the multivariate analysis. Third, we assessed the use of concomitant medications at the moment of ABI initiation, therefore we cannot be absolutely positive that the concurrent therapies for comorbidities have been used for the entire ABI treatment. Finally, although we report a positive trend towards longer OS in men receiving ASi, this should be interpreted with caution, since patients’ subsequent therapies were considerably heterogeneous, which could have a crucial effect on OS. Issues, which are presented in our study, merit further investigations in prospective trials with larger populations.

## Conclusions

To our knowledge, this is the first study demonstrating clinical benefits of ASi among post-chemotherapy mCRPC patients during ABI treatment. In our study population, men who received ASi, had statistically longer duration of ABI therapy versus those without such therapy. The use of ASi may potentially be considered as a new, non-oncological, predictive indicator for longer ABI therapy. Our results, although based on a small sample size, shed a new light on the usefulness of ASi among PC patients. Further cardio-oncology research in the form of prospective clinical trials are needed to confirm these observations. 

## Data Availability Statement

The data sets analyzed during the current study are not publicly available due to the Polish National Health Fund Drug Program policy. Requests to access these datasets should be directed to MW, michal.wilk@ecz-otwock.pl.

## Ethics Statement

The studies involving human participants were reviewed and approved by Bioethical Committee at the Centre of Postgraduate Medical Education in Poland. The patients/participants provided their written informed consent to participate in this study.

## Author Contributions

Design and conception: MW and SS. Patient recruitment: MW, AW-G, and IS. Data collection and analyses: MW. Writing of manuscript: MW and SS. Editing and reviewing the manuscript: MW, A-WG, IS, and SS. Final approval of the version to be published: MW, A-WG, IS, and SS. All authors contributed to the article and approved the submitted version.

## Conflict of Interest

IS reports receiving research grants (not related to this study) and lecture fees from Janssen. SS reports receiving lecture fee from Janssen, unrelated to the subject of this study.

The remaining authors declare that the research was conducted in the absence of any commercial or financial relationships that could be construed as a potential conflict of interest.

## References

[1] Siegel RL Miller KD Jemal A. Cancer statistics, 2020. CA Cancer J Clin (2020) 70:7–30. 10.3322/caac.21590 31912902

[2] Malvezzi M Carioli G Bertuccio P Boffetta P Levi F La Vecchia C. European cancer mortality predictions for the year 2019 with focus on breast cancer. Ann Oncol (2019) 30:781–7. 10.1093/annonc/mdz051 30887043

[3] O’Donnell A Judson I Dowsett M Raynaud F Dearnaley D Mason M. Hormonal impact of the 17alpha-hydroxylase/C(17,20)-lyase inhibitor abiraterone acetate (CB7630) in patients with prostate cancer. Br J Cancer (2004) 90:2317–25. 10.1038/sj.bjc.6601879 PMC2409523 15150570

[4] Wilk M Waśko-Grabowska A Szmit S. Cardiovascular Complications of Prostate Cancer Treatment. Front Pharmacol (2020) 11:1828. 10.3389/fphar.2020.555475 PMC7783464 33414715

[5] Van Hemelrijck M Garmo H Holmberg L Ingelsson E Bratt O Bill-Axelson A. Absolute and relative risk of cardiovascular disease in men with prostate cancer: results from the Population-Based PCBaSe Sweden. J Clin Oncol (2010) 28:3448–56. 10.1200/JCO.2010.29.1567 20567006

[6] Epstein MM Edgren G Rider JR Mucci LA Adami H-O. Temporal trends in cause of death among Swedish and US men with prostate cancer. J Natl Cancer Inst (2012) 104:1335–42. 10.1093/jnci/djs299 PMC3529593 22835388

[7] Davis MK Rajala JL Tyldesley S Pickles T Virani SA. The Prevalence of Cardiac Risk Factors in Men with Localized Prostate Cancer Undergoing Androgen Deprivation Therapy in British Columbia, Canada. J Oncol (2015) 2015:e820403. 10.1155/2015/820403 PMC4537764 26300918

[8] Oken MM Creech RH Tormey DC Horton J Davis TE McFadden ET. Toxicity and response criteria of the Eastern Cooperative Oncology Group. Am J Clin Oncol (1982) 5:649–55. 10.1097/00000421-198212000-00014 7165009

[9] Reid IA Morris BJ Ganong WF. The Renin-Angiotensin System. Annu Rev Physiol (1978) 40:377–410. 10.1146/annurev.ph.40.030178.002113 205167

[10] George AJ Thomas WG Hannan RD. The renin-angiotensin system and cancer: old dog, new tricks. Nat Rev Cancer (2010) 10:745–59. 10.1038/nrc2945 20966920

[11] Uemura H Hoshino K Kubota Y. Engagement of renin-angiotensin system in prostate cancer. Curr Cancer Drug Targets (2011) 11:442–50. 10.2174/156800911795538101 21395553

[12] Domińska K Okła P Kowalska K Habrowska-Górczyńska DE Urbanek KA Ochędalski T. Angiotensin 1–7 modulates molecular and cellular processes central to the pathogenesis of prostate cancer. Sci Rep (2018) 8:15772. 10.1038/s41598-018-34049-8 30361641 PMC6202343

[13] Woo Y Jung Y-J. Angiotensin II receptor blockers induce autophagy in prostate cancer cells. Oncol Lett (2017) 13:3579–85. 10.3892/ol.2017.5872 PMC5431597 28529582

[14] Ronquist G Frithz G Wang Y-H Lindeborg T. Captopril may reduce biochemical (prostate-specific antigen) failure following radical prostatectomy for clinically localized prostate cancer. Scand J Urol Nephrol (2009) 43:32–6. 10.1080/00365590802468875 18932051

[15] Uemura H Hasumi H Kawahara T Sugiura S Miyoshi Y Nakaigawa N. Pilot study of angiotensin II receptor blocker in advanced hormone-refractory prostate cancer. Int J Clin Oncol (2005) 10:405–10. 10.1007/s10147-005-0520-y 16369744

[16] Siltari A Murtola TJ Talala K Taari K Tammela TLJ Auvinen A. Antihypertensive drug use and prostate cancer-specific mortality in Finnish men. PloS One (2020) 15 (6): e0234269. 10.1371/journal.pone.0234269 32598349 PMC7323967

[17] Attard G Reid AHM Auchus RJ Hughes BA Cassidy AM Thompson E. Clinical and biochemical consequences of CYP17A1 inhibition with abiraterone given with and without exogenous glucocorticoids in castrate men with advanced prostate cancer. J Clin Endocrinol Metab (2012) 97:507–16. 10.1210/jc.2011-2189 22170708

[18] Kwok T Ohlsson C Vandenput L Tang N Zhang Y Tomlinson B. ACE inhibitor use was associated with lower serum dehydroepiandrosterone concentrations in older men. Clin Chim Acta (2010) 411:1122–5. 10.1016/j.cca.2010.04.011 PMC2883618 20403346

[19] Attard G Reid AHM Yap TA Raynaud F Dowsett M Settatree S. Phase I clinical trial of a selective inhibitor of CYP17, abiraterone acetate, confirms that castration-resistant prostate cancer commonly remains hormone driven. J Clin Oncol (2008) 26:4563–71. 10.1200/JCO.2007.15.9749 18645193

[20] Titus MA Schell MJ Lih FB Tomer KB Mohler JL. Testosterone and dihydrotestosterone tissue levels in recurrent prostate cancer. Clin Cancer Res (2005) 11:4653–7. 10.1158/1078-0432.CCR-05-0525 16000557

[21] Fiandalo MV Stocking JJ Pop EA Wilton JH Mantione KM Li Y. Inhibition of dihydrotestosterone synthesis in prostate cancer by combined frontdoor and backdoor pathway blockade. Oncotarget (2018) 9:11227–42. 10.18632/oncotarget.24107 PMC5834294 29541409

[22] Perachino M Cavalli V Bravi F. Testosterone levels in patients with metastatic prostate cancer treated with luteinizing hormone-releasing hormone therapy: prognostic significance? BJU Int (2010) 105:648–51. 10.1111/j.1464-410X.2009.08814.x 19747358

[23] DeLong M Logan JL Yong K-C Lien Y-HH. Renin–angiotensin blockade reduces serum free testosterone in middle-aged men on haemodialysis and correlates with erythropoietin resistance. Nephrol Dial Transplant (2005) 20:585–90. 10.1093/ndt/gfh638 15735242

[24] Koshida H Takeda R Miyamori I. Lisinopril decreases plasma free testosterone in male hypertensive patients and increases sex hormone binding globulin in female hypertensive patients. Hypertens Res (1998) 21:279–82. 10.1291/hypres.21.279 9877521

[25] Grönroos PE Irjala KM Vesalainen RK Kantola IM Leinonen VM Helenius TI. Effects of ramipril on the hormone concentrations in serum of hypertensive patients. Eur J Clin Chem Clin Biochem (1997) 35:411–4. 10.1515/cclm.1997.35.6.411 9228322

[26] Szmit S Zaborowska M Waśko-Grabowska A Żołnierek J Nurzyński P Filipiak KJ. Cardiovascular comorbidities for prediction of progression-free survival in patients with metastatic renal cell carcinoma treated with sorafenib. Kidney Blood Press Res (2012) 35:468–76. 10.1159/000338175 22688785

[27] Lu-Yao G Nikita N Keith SW Nightingale G Gandhi K Hegarty SE. Mortality and Hospitalization Risk Following Oral Androgen Signaling Inhibitors Among Men with Advanced Prostate Cancer by Pre-existing Cardiovascular Comorbidities. Eur Urol (2019) 77 (2):158–66. 10.1016/j.eururo.2019.07.031 PMC6980462 31420248

[28] Munger MA. Use of Angiotensin Receptor Blockers In Cardiovascular Protection. P T (2011) 36:22–40. 21386934 PMC3046622

[29] Heart Outcomes Prevention Evaluation Study Investigators Yusuf S Sleight P Pogue J Bosch J Davies R. Effects of an angiotensin-converting-enzyme inhibitor, ramipril, on cardiovascular events in high-risk patients. N Engl J Med (2000) 342:145–53. 10.1056/NEJM200001203420301 10639539

[30] Dahlöf B Devereux RB Kjeldsen SE Julius S Beevers G de Faire U. Cardiovascular morbidity and mortality in the Losartan Intervention For Endpoint reduction in hypertension study (LIFE): a randomised trial against atenolol. Lancet (2002) 359:995–1003. 10.1016/S0140-6736(02)08089-3 11937178

[31] Jacoby DS Rader DJ. Renin-angiotensin system and atherothrombotic disease: from genes to treatment. Arch Intern Med (2003) 163:1155–64. 10.1001/archinte.163.10.1155 12767951

[32] Chen S Acou W-J Kiuchi MG Meyer C Sommer P Martinek M. Association of Preoperative Renin-Angiotensin System Inhibitors With Prevention of Postoperative Atrial Fibrillation and Adverse Events: A Systematic Review and Meta-analysis. JAMA Netw Open (2019) 2:e194934. 10.1001/jamanetworkopen.2019.4934 31150082 PMC6547087

[33] Sharma P Hakimian S Jobanputra Y Peles S Byron B. Prevention of chemo-induced cardiotoxicity with ACE inhibitors and angiotensin receptor blockers. JCO (2018) 36:e14513–3. 10.1200/JCO.2018.36.15_suppl.e14513

[34] Długosz-Danecka M Gruszka AM Szmit S Olszanecka A Ogórka T Sobociński M. Primary Cardioprotection Reduces Mortality in Lymphoma Patients with Increased Risk of Anthracycline Cardiotoxicity, Treated by R-CHOP Regimen. Chemotherapy (2018) 63:238–45. 10.1159/000492942 30372698

[35] Szmit S Langiewicz P Złnierek J Nurzyński P Zaborowska M Filipiak KJ. Hypertension as a predictive factor for survival outcomes in patients with metastatic renal cell carcinoma treated with sunitinib after progression on cytokines. Kidney Blood Press Res (2012) 35:18–25. 10.1159/000329933 21849795

[36] Arafat HA Gong Q Chipitsyna G Rizvi A Saa CT Yeo CJ. Antihypertensives as novel antineoplastics: angiotensin-I-converting enzyme inhibitors and angiotensin II type 1 receptor blockers in pancreatic ductal adenocarcinoma. J Am Coll Surg (2007) 204:996–1005; discussion 1005-1006. 10.1016/j.jamcollsurg.2007.01.067 17481528

